# Inducer-free recombinant protein production in *Trichoderma reesei*: secretory production of endogenous enzymes and heterologous nanobodies using glucose as the sole carbon source

**DOI:** 10.1186/s12934-023-02109-y

**Published:** 2023-05-19

**Authors:** Toshiharu Arai, Mayumi Wada, Hiroki Nishiguchi, Yasushi Takimura, Jun Ishii

**Affiliations:** 1 Biological Science Research, Kao Corporation, 1334 Minato, Wakayama, 640‑8580 Japan; 2 Graduate School of Science, Technology and Innovation, Kobe University, 1-1 Rokkodai, Nada, Kobe, 657-8501 Japan; 3 Engineering Biology Research Center, Kobe University, 1-1 Rokkodai, Nada, Kobe, 657-8501 Japan

**Keywords:** *Trichoderma reesei*, Recombinant protein, Cellulase, Cellulose, Glucose, Antibody, V_HH_, Nanobody, XYR1, ACE3

## Abstract

**Background:**

The filamentous fungus *Trichoderma reesei* has been used as a host organism for the production of lignocellulosic biomass-degrading enzymes. Although this microorganism has high potential for protein production, it has not yet been widely used for heterologous recombinant protein production. Transcriptional induction of the cellulase genes is essential for high-level protein production in *T. reesei*; however, glucose represses this transcriptional induction. Therefore, cellulose is commonly used as a carbon source for providing its degraded sugars such as cellobiose, which act as inducers to activate the strong promoters of the major cellulase (cellobiohydrolase 1 and 2 (*cbh1* and *cbh2*) genes. However, replacement of *cbh1* and/or *cbh2* with a gene encoding the protein of interest (POI) for high productivity and occupancy of recombinant proteins remarkably impairs the ability to release soluble inducers from cellulose, consequently reducing the production of POI. To overcome this challenge, we first used an inducer-free biomass-degrading enzyme expression system, previously developed to produce cellulases and hemicellulases using glucose as the sole carbon source, for recombinant protein production using *T. reesei*.

**Results:**

We chose endogenous secretory enzymes and heterologous camelid small antibodies (nanobody) as model proteins. By using the inducer-free strain as a parent, replacement of *cbh1* with genes encoding two intrinsic enzymes (aspartic protease and glucoamylase) and three different nanobodies (1ZVH, caplacizumab, and ozoralizumab) resulted in their high secretory productions using glucose medium without inducers such as cellulose. Based on signal sequences (carrier polypeptides) and protease inhibitors, additional replacement of *cbh2* with the nanobody gene increased the percentage of POI to about 20% of total secreted proteins in *T. reesei*. This allowed the production of caplacizumab, a bivalent nanobody, to be increased to 9.49-fold (508 mg/L) compared to the initial inducer-free strain.

**Conclusions:**

In general, whereas the replacement of major cellulase genes leads to extreme decrease in the degradation capacity of cellulose, our inducer-free system enabled it and achieved high secretory production of POI with increased occupancy in glucose medium. This system would be a novel platform for heterologous recombinant protein production in *T. reesei*.

**Supplementary Information:**

The online version contains supplementary material available at 10.1186/s12934-023-02109-y.

## Background

Recombinant proteins, which have been used as industrial enzymes, have recently been shown to have potential as advanced materials (e.g., spider silk) [1] and alternative edible proteins [2] (e.g., milk whey and egg proteins [3]). Furthermore, owing to their highly specific binding properties, antibodies have multiple applications, including biological therapy (e.g., treatment of breast cancer and acute lymphocytic leukemia) [4], lateral flow tests, immunological assays (e.g., detection of SARS-CoV-2, pregnancy, and toxic substances in food) [5], and cell and tissue imaging (e.g., microscopy observation and high-content screening) [6]. However, the high cost of protein production hinders the commercialization of antibodies and other highly functional biomaterials. Therefore, efforts are being made to improve both the valuable proteins themselves and the hosts used for their production [7, 8, 9].

Bispecific T-cell engagers (BiTE; blinatumomab) [10] and small antibody fragments, such as single-chain variable fragments (scFv) and antigen-binding fragments (Fab), have recently attracted attention as components for the development of next-generation antibodies [11]. Additionally, the variable domain of heavy-chain antibodies (V_HH_; also known as nanobodies) derived from camelid single-domain antibodies (sdAbs) are promising for use as small antibody fragments [12, 13]. Nanobodies have a simple structure with only a heavy chain and no light chain; therefore, they are among the smallest functional antibody fragments. Because of this feature, nanobodies are suitable for the construction of bispecific or bivalent/trivalent tandem repeat antibodies [14]. Other important characteristics of nanobodies are their low molecular weight, high resistance to proteolysis and thermal denaturation, high solubility, and ease of production by microorganisms [15, 16]. In contrast to full-length immunoglobulin G antibodies, which are typically produced in Chinese hamster ovary cells, small antibody fragments such as scFv and nanobodies (especially the sdAb nanobody) are more suitable for microbial production and have great potential to reduce production costs [17].

Bacteria and yeasts are often used as platforms for the microbial production of recombinant proteins [7, 18]. The filamentous fungus *Trichoderma reesei* (syn. *Hypocrea jecorina*) is an attractive host cell candidate for recombinant protein production [19]. *T. reesei* has been bred for many years as a host microorganism for the industrial production of high levels of cellulosic biomass-degrading enzymes [20, 21, 22]. Owing to its high potential for secreted protein production, *T. reesei* has been the focus of research efforts to produce recombinant proteins other than lignocellulolytic enzymes such as cellulases and xylanases [19, 23, 24, 25].

**Fig. 1:**
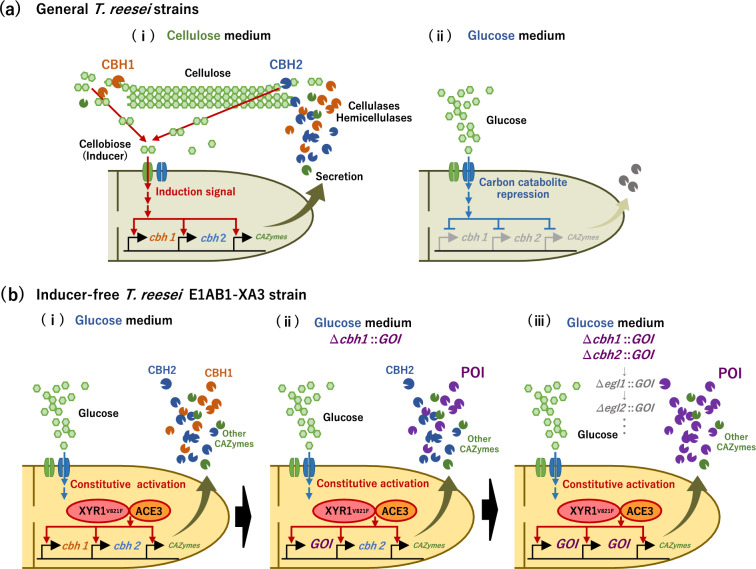
Schematic representation of protein production systems using *Trichoderma reesei*. **a** In *T. reesei* strains, (i) cellulose-derived sugars (such as cellobiose) released by cellobiohydrolase (CBH)1 and CBH2 induce the production of large amounts of cellulases and hemicellulases. (ii) When glucose is used as the carbon source, the production of cellulase and hemicellulase is suppressed by carbon catabolite repression (CCR). **b** In inducer-free *T. reesei* E1AB1-XA3 strain, with release from CCR and modification of the transcription factors, (i) it is possible to produce cellulases and hemicellulases using only glucose as the carbon source. As a strategy for heterologous protein production, (ii) the most abundant native cellulase, CBH1, is replaced with the target protein, and (iii) high purity and high production are possible by replacing CBH2 with other CAZymes

However, *T. reesei* is not commonly used as a host organism for recombinant protein production. A major limitation of *T. reesei* as a host for recombinant protein production is that high protein production generally requires the use of a natural and specialized inducible cellulase expression system. Specifically, the expression of the major cellulases cellobiohydrolases 1 and 2 (CBH1 and CBH2), whose own genes are under the control of this transcriptional induction system, is required for the release of cellulose-derived sugars (such as cellobiose) as direct inducers (Fig. 1a-i). Induced cellulase expression results in the release of considerable amounts of inducers through the degradation of cellulose [26]. Under inducible conditions with cellulose as the carbon source, substantially high amounts of cellulases and hemicellulases are secreted into the culture medium, with production exceeding 100 g/L under appropriate conditions [20, 21, 22]. Four major cellulases (CBH1, CBH2, and endoglucanases 1 and 2 [EGL1 and EGL2]) account for more than 90% of the total secreted proteins in *T. reesei* [27], of which 60% and 20% are CBH1 and CBH2, respectively [28]. A potential approach for recombinant protein production in *T. reesei* is to use the strongest promoter, *cbh1* [29, 30].

For increased production and purity (POI occupancy in total secreted proteins) of recombinant proteins in *T. reesei*, two major cellulase genes, *cbh1* and/or c*bh2*, should be replaced with a gene encoding the protein of interest (POI) (gene of interest, GOI). However, the knockout of *cbh1* and/or *cbh2* markedly impairs the ability of *T. reesei* to break down cellulose and release soluble inducers, resulting in growth reduction and induction defects [31, 32]. Therefore, the use of *cbh* promoters for recombinant protein production in *T. reesei* requires either retaining the major native cellulases to release the inducers from expensive, insoluble cellulose [30], or using more expensive, soluble disaccharides as direct inducers (cellobiose, sophorose, lactose, etc.) [25]. Although the use of a soluble, inexpensive, and common carbon source (i.e., glucose) is preferable in terms of cost reduction, the expression of cellulase and hemicellulose is drastically suppressed by carbon catabolite repression (CCR) [33]. Therefore, the use of glucose as a carbon source prevented the activity of the natural cellulase-inducible expression system, and the major cellulase promoters could not be used for protein production (Fig. 1a-ii).

We previously developed an alternative strategy for recombinant protein production in *T. reesei* using an inducer-free transcription activation system for the simple and cost-effective production of endogenous biomass-degrading enzymes [34]. The engineered inducer-free strain produced high levels of cellulase and hemicellulase using inexpensive glucose as the sole carbon source without cellulose or any other sugars as inducers (Fig. 1b, E1AB1-XA3 strain). This strain is based on the high enzyme-producing mutant E1AB1 [35] with multiple modifications for the transcriptional regulators of cellulase and hemicellulase genes: deletion of transcriptional repressors (∆*ace1* and ∆*rce1*) and constitutive expression of the major transcriptional activators (mutated XYR1^V821F^ and partially truncated ACE3) (Fig. 1b-i). Owing to the presence of strong transcriptional activators in this strain, induction by cellulose degradation is not required. Considering that the engineered strain is promising for high productivity and occupancy of recombinant proteins, we aimed to utilize the strong *cbh* promoter of this strain by replacing *cbh1* and *cbh2* with GOI to express the POI.

In the present study, we used an inducer-free cellulase/hemicellulase expression system in *T. reesei* for recombinant protein production using glucose (the most commonly used carbon source) instead of cellulose. As a proof of concept, we initially replaced *cbh1*, which encodes one of the major native cellulases, with the POI genes (GOIs) using the inducer-free E1AB1-XA3 strain as the parent strain (Fig. 1b-ii). Two intrinsic secretory enzymes and three camelid nanobodies were selected for the production of endogenous and heterologous proteins, respectively, which showed high secretory production in glucose medium lacking cellulose or other inducers. Based on the types of signal sequences (with or without a carrier polypeptide and cleavage linker) and the addition of protease inhibitors, additional replacement of *cbh2* with the bivalent nanobody gene as the heterologous POI further increased the productivity and occupancy of the POI in the total secreted proteins in *T. reesei* (Fig. 1b-iii). Our approach provides new insights into the application of *T. reesei* as a microbial platform for low-cost, highly secretory recombinant protein production using glucose as the sole carbon source. Furthermore, this strategy can resolve the challenges associated with specialized transcription activation mechanisms and the need to use the relatively expensive and complicated carbon source cellulose.

## Results

### Proof of concept for inducer-free recombinant protein production using endogenous secretory enzymes in *T. reesei*

**Fig. 2:**
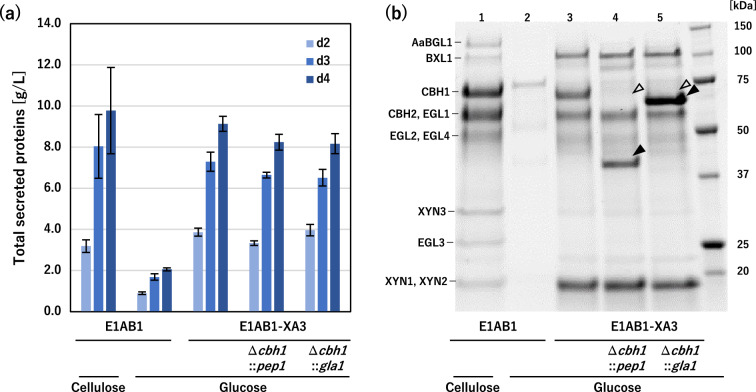
Endogenous secreted protease and glucoamylase production from *T. reesei* using an inducer-free system. *T. reesei* E1AB1 strain was cultivated in shake flasks on an inducing medium containing 3% cellulose and a non-inducing medium containing 3% glucose as a carbon source. E1AB1-XA3, E1AB1-XA3Δ*cbh1*::*pep1* (tre74156, aspartic protease) and E1AB1-XA3Δ*cbh1*::*gla1* (tre1885, glucoamylase) strains were cultivated on a non-inducing medium containing 3% glucose. **a** Total secreted proteins after 2, 3, and 4 days of cultivation. **b** SDS-PAGE analysis of the secreted proteins, the supernatant after 3 days of cultivation were diluted 8-fold and loaded with 5 µL. Open arrowhead indicates the deleted protein, corresponding to the CBH1. Closed arrowheads indicate clearly overexpressed proteins, corresponding to protease and glucoamylase. Error bars indicate standard deviations

Initially, the production of cellulase and hemicellulase of the conventional (non-inducer-free) E1AB1 strain was evaluated both in usual (cellulose) and inducer-free (glucose) conditions. While the E1AB1 strain secreted large amounts of cellulase and hemicellulase in the culture medium when cellulose was used as the carbon source (Fig. 2a, b-lane 1), the same strain produced little of them when glucose was used as the carbon source (Fig. 2a, b-lane 2). In contrast, as shown in our previous study [34], the inducer-free E1AB1-XA3 strain produced major cellulases and hemicellulases when cultured in glucose medium (inducer-free conditions), with a comparable level of production to the E1AB1 strain (Fig. 2a, b-lane 3).

As a proof of concept for recombinant protein production using the inducer-free transcription activation system, we replaced the major native cellulase *cbh1* with a GOI encoding the endogenous secretory enzymes of *T. reesei* in the E1AB1-XA3 strain. Aspartic protease PEP1 (gene ID: tre74156) [36] and glucoamylase GLA1 (gene ID: tre1885) [37] were selected as model recombinant proteins of endogenous enzymes. Homologous recombination of these genes was performed at the *cbh1* genomic locus in the E1AB1-XA3 strain, and protein production was tested using glucose as the carbon source.

When the E1AB1-XA3 strains harboring *pep1* and *gla1* were cultivated in glucose media, there was no significant change in the total amount of secreted proteins compared to the parental E1AB1-XA3 strain (Fig. 2a). However, the disappearance of the CBH1 protein (Fig. 2b, open triangles) and the appearance of distinct band patterns corresponding to PEP1 and GLA1 proteins (Fig. 2b, solid triangles) were observed in the recombinant E1AB1-XA3 strains, respectively. Thus, the inducer-free *T. reesei* E1AB1-XA3-based recombinant strains, lacking the secretory expression of the dominant CBH1 protein, enabled recombinant protein production of endogenous enzymes using a strong *cbh1* promoter in glucose media.

### Inducer-free secretory production of three nanobodies as heterologous proteins in *T. reesei*

**Fig. 3:**
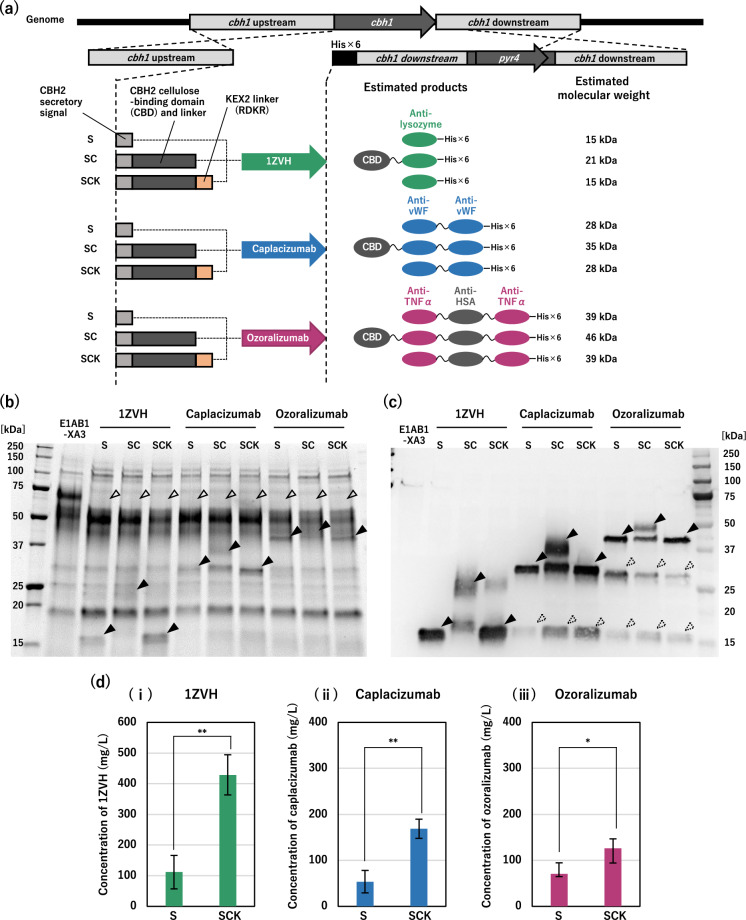
Inducer-free nanobodies production by single replacement of cbh1 with three expression patterns of nanobody genes. **a** Configuration of the nanobody expression cassettes. Each nanobody was combined with the secretory signal peptide of CBH2 (S), the secretory signal peptide and the cellulose-binding domain (CBD) (SC), and the cleavage site of the KEX2 protease between the CBD and the nanobody (SCK). A 6× His-tag sequence was added to the C-terminus of each nanobody. Homologous recombination of the *cbh1* locus was performed in the E1AB1-XA3 strain. *T. reesei* E1AB1-XA3 strain and transformants were cultivated in shake flasks on non-inducing medium containing 3% glucose. **b** SDS-PAGE analysis of the secreted proteins, the supernatant after 2 days of cultivation were diluted 3-fold and loaded with 5 µL. Open triangles indicate deleted proteins corresponding to CBH1. The closed triangles indicate the target nanobody, as estimated from the molecular weight. **c** Western blot analysis using an anti-His tag antibody (Fig. 3b). The closed triangles indicate the target nanobody, as estimated from the molecular weight. Dashed triangles indicate cleaved nanobodies. **d** Concentration of nanobodies secreted into the culture supernatant on day 4. The concentration of the nanobodies was determined by western blotting using the purified nanobody as a calibration curve. Error bars indicate standard deviation. Statistical significance was determined using two-tailed unpaired Student’s *t*-test. *p < 0.05. **p < 0.01

To further demonstrate the applicability of the inducer-free expression system for secretory production of heterologous proteins, we selected three nanobodies as production models (Fig. 3a). The first was a monovalent antilysozyme nanobody with a PDB ID registered as 1ZVH [38, 39]. The second is a bivalent nanobody, caplacizumab, which is a tandemly repeated V_HH_ for the anti-von Willebrand factor (vWF) linked via a trialanine linker that has been approved for the treatment of acquired thrombotic thrombocytopenic purpura (aTTP) [40, 41]. The third is a trivalent bispecific nanobody, ozoralizumab, which is linked via a Gly-Ser linker with two V_HH_ fragments for anti-tumor necrosis factor alpha (TNF-α) flanking each side of a V_HH_ fragment for anti-human serum albumin (HSA) and has been approved as a biologic for the treatment of rheumatoid arthritis [42]. These nanobodies, including bivalent and trivalent proteins with multiple V_HH_ fragments linked by flexible linkers, were selected to verify the differences in the levels of protein production and degradation compared to monovalent nanobodies in *T. reesei*.

Additionally, we tested the expression of three different forms of secretory proteins for each nanobody. The first is the most common strategy, involving the addition of a secretory signal peptide of CBH2 (Fig. 3a, S) [43]. The second is to fuse the cellulose-binding domain (CBD) and linker of CBH2 with the secretory signal sequence as a carrier polypeptide (Fig. 3a, SC), which has been reported as a strategy for improving the secretory production of heterologous proteins in filamentous fungi [44]. The third was a slightly modified version of the method described above, in which the fused carrier polypeptide (including the secretory signal) was cleaved by an endogenous protease [44]. In this study, a cleavage site of the KEX2 protease (RDKR; KEX2 linker) was inserted between the CBD and nanobody to cleave it in the Golgi apparatus (Fig. 3a, SCK). The expression constructs for each nanobody were designed to add a histidine-tag (His-tag) sequence at the C-terminus, and homologous recombination into the *cbh1* locus was performed in the E1AB1-XA3 strain.

Inducer-free strains expressing either expression construct did not significantly reduce the amount of total secreted proteins in the supernatant cultured in glucose medium (Additional file 1: Fig. S1). As shown through SDS-PAGE for the secretory production of endogenous enzymes (Fig. 2), all recombinant strains appeared as new protein bands at positions estimated to have roughly the same molecular weight as each nanobody construct (solid triangles), along with the loss of CBH1 protein (open triangles) (Fig. 3b). For more visible confirmation of recombinant protein expression, western blot analysis with an anti-His tag antibody was performed (Fig. 3c). The addition of CBH2 secretion signal peptide (S) was found to be effective in the secretory production of all three nanobodies, while undesired (non-target) protein bands, presumably cleaved around the linker connecting the multiple V_HH_, were also observed at the approximately monovalent or bivalent size position for caplacizumab and ozoralizumab (dashed triangles) (Fig. 3c, S). In the case of nanobodies with the CBH2 secretory signal and CBD-carrier polypeptide (SC, without KEX2 linker), bands were observed at the positions where the correct CBD-nanobody fusion proteins presumably expressed (Fig. 3c, SC, solid triangles), while other bands were also observed at slightly lower positions (SC, no triangles). These lower bands however exhibited slightly different (higher) sizes from the bands of the nanobodies adding with secretory signal (S, solid triangles), suggesting that the CBD-carrier polypeptide (SC) may be cleaved in the middle of CBD-linker of CBH2 by an endogenous protease. In contrast, the nanobodies harboring the CBH2 secretory signal and CBD-carrier polypeptide along with the KEX2 linker (SCK) displayed similar band sizes with relatively higher productivities compared to those with the CBH2 secretory signal (S) (Fig. 3c). As quantified by western blotting, the secretory production levels of nanobodies with the signal, CBD, and KEX2 linker (SCK) were 3.85-fold (Fig. 3d-i), 3.14-fold (Fig. 3d-ii), and 1.79-fold (Fig. 3d-iii) higher than those with the secretory signal sequence (S) for 1ZVH, caplacizumab, and ozoralizumab, respectively.

These results indicated that the inducer-free transcription activation system of *T. reesei* [34] resulted in the replacement of *cbh1* in the genome with a GOI for the secretory production of heterologous recombinant proteins in the glucose culture medium. Additionally, the use of the CBD carrier polypeptide and KEX2 linker along with the CBH2 secretory signal sequence [44] improved the production levels of heterologous recombinant proteins (nanobodies) in the inducer-free E1AB1-XA3 strain.

### Production of nanobodies adding with protease inhibitors

In the secretory production of recombinant proteins, degradation of recombinant proteins, including antibodies, by extracellular proteases is an issue that could often occur in their secretory production [45]. In fact, bands of presumed degradation products were detected in the secretory production of bivalent and trivalent nanobodies (Fig. 3c). Therefore, in order to simply examine the possibility of inhibiting protease degradation, we evaluated the effect of a cocktail of serine and cysteine protease inhibitors on the degradation of nanobodies. Because of the highest productivity (Fig. 2), the expression pattern with SCK polypeptide was adopted for inducer-free cultures using 3% glucose as the carbon source.

**Fig. 4:**
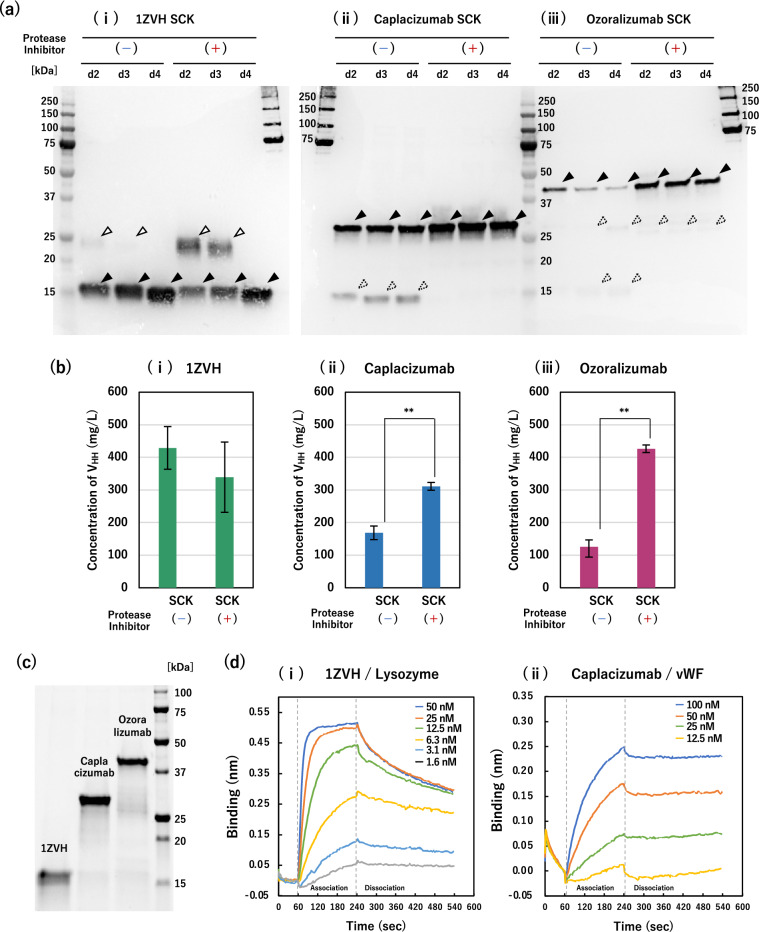
Nanobody production with protease inhibition and functional evaluation. *T. reesei* E1AB1-XA3 strain based transformants were cultivated in shake flasks on a non-inducing medium containing 3% glucose and with (+)/without (−) protease inhibitor cocktail. **a** Western blotting analysis using anti-His tag antibody on gels that were performed on SDS-PAGE loaded with 5 µL of 8-fold diluted supernatant. Closed triangles indicate the target nanobody as estimated from the molecular weight. Open triangles indicate the un-cleaved protein at the KEX2 linker, corresponding to the CBD-1ZVH fusion protein. Dashed triangles indicate cleaved nanobodies. **b** Concentration of nanobodies secreted to culture supernatant at day 4. The nanobodies concentration were determined by western blotting using the purified nanobody as a calibration curve. **c** Purified His-tagged nanobodies using Ni-NTA beads. Protease inhibitor-added culture supernatant from day 4 were used for purification. **d** Kinetics analysis of 1ZVH/lysozyme and caplacizumab/vWF. Association and dissociation rate were measured by reacting the sensor with nanobody conjugated via His-tag and various concentrations of antigen. Error bars indicate standard deviations. Statistical significance was determined by a two-tailed unpaired Student’s *t*-test. *p < 0.05. **p < 0.01

All strains producing three nanobodies reduced little the amounts of total secreted proteins in the supernatant by the addition of protease inhibitors (Additional file 1: Fig. S2). For the production of monovalent 1ZVH (Fig. 4a-i), western blot analysis of His-tagged proteins showed that the intensity of target bands (solid triangles) gradually increased over the days of cultivation under both conditions with and without protease inhibitors. On the other hand, clear bands with higher molecular weight (open triangles) were observed on day 2 and 3 in the cultures with protease inhibitors (Fig. 4a-i, +). The bands of the same sizes, albeit in small amounts, were also observed on day 2 or 3 in the inhibitor-lacking condition (Fig. 4a-i, –). These higher bands were estimated to be the CBD-1ZVH fusion protein that has not cleaved at the KEX2 linker. To confirm this, an adsorption test on cellulose was performed. After adsorption of purified proteins on cellulose, the lower bands presumed to be 1ZVH remained, while the higher bands presumed to be CBD-1ZVH disappeared (Additional file 1: Fig. S3). This suggested that the higher bands contained CBD and were probably the CBD-1ZVH fusion protein that has not yet been cleaved by the KEX2 protease. Note that, however, after 4 days of cultivation, the higher bands completely disappeared and only the lower bands remained both in the inhibitor-adding and -lacking conditions, thereby indicating that the 1ZVH infallibly cleaving the carrier polypeptide (CBD-KEX2 linker) was produced (Fig. 4ai). For the case of monovalent 1ZVH, there was no positive effect to the addition of protease inhibitors on protein productivity.

In contrast, the addition of protease inhibitors decreased the band intensities of the degraded products (dashed triangles) and increased those of the target nanobodies for the cases in the production of bivalent caplacizumab (Fig. 4a-ii) and trivalent ozoralizumab (Fig. 4a-iii). In particular, the target band intensities of trivalent ozoralizumab decreased with the days of cultivation in the absence of protease inhibitors compared to those maintained in the presence of inhibitors (Fig. 4a-iii). In addition, no bands of distinct CBD-fusion proteins due to the defective cleavage at the KEX2 site were detected (Fig. 4a-ii, iii). Quantification of produced nanobodies showed 1.85-fold (Fig. 4b-ii) and 3.38-fold (Fig. 4b-iii) increases in the production of caplacizumab and ozoralizumab, respectively, compared with the conditions lacking protease inhibitors.

As demonstrated above, the inhibition of protease activities on the production of nanobodies was important, especially for bivalent and trivalent forms linked by flexible linkers, in terms of improving both the productivity and quality of the products. In contrast, the production of 1ZVH indicated that the cleavage of CBD-carrier polypeptide by the KEX2 protease might have depended on the sequence or size of the target proteins.

### Confirmation of binding abilities of nanobodies produced in *T. reesei*

The His-tagged nanobodies were purified from the culture supernatant on the 4 days of cultivation with protease inhibitors using nickel-nitrilotriacetic acid (Ni-NTA) resin. Analysis by SDS-PAGE showed that the purified nanobodies appeared as almost single bands with the desired sizes (Fig. 4c). The purified nanobodies were then evaluated for their binding affinities to each corresponding antigen using a BLItz system (ForteBio) based on biolayer interferometry [46]. For trivalent ozoralizumab, the TNF-α antigen is small at 17 kDa [47] compared to the ozoralizumab antibody (38 kDa) [42], which can be immobilized on the sensor via a His-tag, making it difficult to accurately analyze the antigen-binding dissociation signal using BLItz. Therefore, we evaluated the binding affinities of 1ZVH and caplacizumab to lysozyme and vWF, respectively. For 1ZVH, the equilibrium dissociation constant between the antibody and its antigen calculated from the association/dissociation kinetics was *K*_D_=1.5 nM (Fig. 4d-i), which was considered to be an appropriate value [38, 39]. For caplacizumab, although the association curve for the antigen was obtained, little dissociation was observed (Fig. 4d-ii), resulting in the *K*_D_ value not being calculated using the BLItz system. This result is in good agreement with, and reflects the fact that caplacizumab has an extremely low equilibrium dissociation constant (*K*_D_=3.76 pM) [41]. Although we could not lead to the exact *K*_D_ value of the caplacizumab produced in *T. reesei*, the nanobodies produced in this study (at least 1ZVH and caplacizumab) were found to be capable of binding to the antigens.

### Additional replacement of *cbh2* with GOI for high productivity and occupancy

To further increase the productivity of secreted nanobodies, we attempted to replace *cbh2* with GOI as well as *cbh1*. Because CBH2 is the second most dominant secreted protein following CBH1, the additional replacement of cbh2 with GOI in the inducer-free strain would not only increase the production of POI but also their occupancy in total secreted proteins. To test this assumption, caplacizumab was chosen and its double replacement strain was constructed. Using the engineered inducer-free E1AB1-XA3 strain to produce the secretory caplacizumab with the SCK polypeptide under the control of *cbh1* promoter as the parent, further homologous recombination of the same gene encoding the caplacizumab with the SCK polypeptide to replace *cbh2* was performed. As the controls, the same recombinations were implemented to the E1AB1 strain, which requires cellulose as the carbon source for induction.

**Fig. 5:**
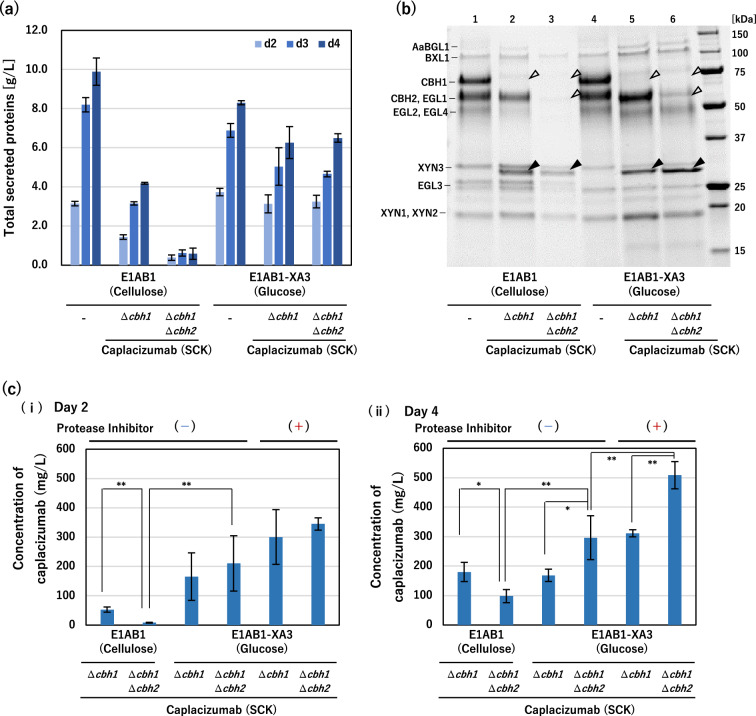
Double replacement of *cbh1* and *cbh2* with caplacizumab for high productivity and purity. Using *T. reesei* E1AB1 and E1AB1-XA3 as parental strains, a single replacement of *cbh1* and double replacement of *cbh1* and *cbh2* with a caplacizumab expression cassette that applied the CBD-carrier polypeptide and KEX2 linker (SCK) pattern was performed. The E1AB1 strain and transformants were cultivated in shake flasks on an induction medium containing 3% cellulose, and *T. reesei* E1AB1-XA3 strain-based transformants were cultivated in shake flasks on a non-induction medium containing 3% glucose. **a** Total secreted proteins after 2, 3, and 4 days of cultivation. **b** SDS-PAGE analysis of the secreted proteins, the supernatant after 2 days of cultivation were diluted 4-fold and loaded with 5 µL. Open triangles indicate deleted proteins corresponding to CBH1 and CBH2. The closed triangles indicate caplacizumab. **c** Concentration of nanobodies secreted into the culture supernatant on days 2 and 4. The concentration of the nanobodies was determined by western blotting using purified caplacizumab as a calibration curve. Error bars indicate standard deviation. Statistical significance was determined using two-tailed unpaired Student’s *t*-test. *p < 0.05. **p < 0.01

In the cellulose-induced production based on the conventional E1AB1 strain, double replacement of *cbh1* and *cbh2 *significantly reduced the productivity of total secreted proteins (Fig. 5a). This implied that the loss of CBH1 and/or CBH2 was accompanied by a significant decrease in the production of major cellulases, resulting in a greatly reduced ability to degrade and utilize cellulose. In contrast, inducer-free production based on the E1AB1-XA3 strain as the parent strain did not show the significant decrease in the productivity of total secreted proteins despite the deletion of both CBH1 and CBH2 (Fig. 5a). The SDS-PAGE analysis showed that the E1AB1-based strain produced markedly less caplacizumab than the E1AB1-XA3-based strain (solid triangles), when both *cbh1* and *cbh2* were replaced (open triangles) (Fig. 5b, after 2 days of cultivation). These results were also confirmed by the western blotting analysis (Additional file 1: Fig. S4).

Table 1 showed the relative proportions of secreted proteins in the supernatants calculated from the SDS-PAGE results. Both in the E1AB1-based and E1AB1-XA3-based strains, the replacement of *cbh1* and *cbh2* increased the relative content of the target protein, caplacizumab, to approximately 20% of total secreted proteins (Table 1), although the E1AB1-based strain had a much lower total amount of secreted proteins than the E1AB1-XA3-based strain (Fig. 5a, b). Consistent with a previous report [31], an increase in the ratio of other native endoglucanases and xylanases was observed following the deletion of CBH1 and CBH2 (Fig. 5b; Table 1).

**Table 1:** Relative proportion of secreted proteins determined by SDS-PAGE analysis (day 2)

	E1AB1			E1AB1-XA3		
	–	ΔCBH1 Caplacizumab	ΔCBH1 ΔCBH2 Caplacizumab	–	ΔCBH1 Caplacizumab	ΔCBH1 ΔCBH2 Caplacizumab
AaBGL1	1.6	1.0	0.6	0.6	0.4	2.0
BXL1	4.5	3.6	4.9	2.0	4.5	5.7
CBH1	26.9	0.0	0.0	31.2	0.0	0.0
CBH2 + EGL1	26.6	24.0	2.5	29.0	34.4	9.8
EGL2 + EGL4	17.7	9.5	1.9	19.6	20.0	26.0
XYN3	4.3	7.8	9.8	2.1	2.0	3.1
Caplacizumab	0.0	12.2	23.2	0.2	9.4	21.1
EGL3	6.3	10.9	17.2	3.2	3.2	5.6
XYN1 + XYN2	5.0	9.1	13.2	4.3	11.9	11.0
others	7.1	21.8	26.7	7.9	14.2	15.6
Total	100.0	100.0	100.0	100.0	100.0	100.0

From the perspective of productivity, the amount of caplacizumab quantified by western blot analysis was also highest for the E1AB1-XA3-based inducer-free strain with double replacement of *cbh1* and *cbh2* both on day 2 and day 4 of cultivation (Fig. 5c). Especially on day 2, the inducer-free E1AB1-XA3-based double replacement strain, which was cultured in glucose medium, showed significantly higher productivity than the E1AB1-based double replacement strain cultured in cellulose medium (Fig. 5c-i). On day 4, the E1AB1-XA3-based double replacement strain exhibited 1.76-fold (296 mg/L) and 1.63-fold (508 mg/L) higher productivity of caplacizumab than the E1AB1-XA3-based *cbh1* single replacement strain in the conditions for the absence and presence of protease inhibitors, respectively (Fig. 5c-ii).

To summarize all the improvements in the E1AB1-XA3-based inducer-free strain, the double replacement of *cbh1* and *cbh2 *with the target nanobody gene, the use of SCK polypeptide (CBH2 secretory signal and CBD-carrier polypeptide with KEX2 linker), and the addition of protease inhibitors could increase the productivity of caplacizumab by 9.49-fold when compared to the single replacement of *cbh1* and the use of CBH2 secretory signal sequence (S) in the absence of protease inhibitors (Fig. 3d-ii (S), 53.6 mg/L).

## Discussion

In this study, we first applied the inducer-free cellulase/hemicellulase expression system of *T. reesei*, which we recently developed [34], for recombinant protein production. Since the system does not require the inducers that are the degradants of cellulose for protein production, it was possible to produce the recombinant proteins using the inexpensive and easy-to-use sugar, glucose, as the sole carbon source while using the powerful cellulase expression promoters for the POI production. In addition, the inducer-free system has also enabled to delete and replace the multiple genomic genes encoding major cellulases with the GOI (demonstrated the double replacement in this study), which was helpful to increase the productivity of recombinant POI and its occupancy (percentage) in total secreted proteins. Thus, our approach could simultaneously resolve the two biggest problems in the *Trichoderma*-based production of recombinant proteins as described above, increasing the productivity of target recombinant proteins in the glucose medium while reducing the contamination of large amounts of native cellulases.

In the conventional (non-inducer-free) systems, the release of sugars from cellulose, mainly disaccharides such as cellobiose, is the inducing factor for cell growth and the upregulation of cellulase genes, thereby resulting in decrease of POI productivity due to the reduced cellulose degradability associated with the disruption (replacement) of major cellulases (Fig. 5c-i, ii). In contrast, since our inducer-free system can promote the production of recombinant POI using glucose as the carbon source, the POI productivity of inducer-free *cbh1* and *cbh2 *double replacement strain was high from the early phase of culture (day 2, Fig. 5c-i), coupled with the results of avoiding negative effects on cell growth (data not shown). Because glucose (3% used in this study) was exhausted by day 3 of culture as described in previous studies [34], the activity of protein production might be reduced in the late phase of batch culture (day 4, Fig. 5c-ii). In the future, it is hopefully expected that the inducer-free E1AB1-XA3-based strain may exhibit an even higher production level of recombinant proteins when combined with a feeding process that continues to supply glucose.

As shown in this study, our inducer-free system could be applied to the production of various proteins, including endogenous enzymes and heterologous small antibodies. The production of nanobodies using various microorganisms as hosts has garnered attention, though there are few reports of high production above several hundred mg/L [48]. Filamentous fungi, particularly *Aspergillus oryzae* and *Aspergillus awamori*, have emerged as notable hosts for nanobody production, although they still showed relatively low yields of only a few tens of mg/L [48]. In another case, using *T. reesei* as a host, there is a report of 150 mg/L murine Fab antibody fragment produced by fusing CBH1-carrier polypeptide in flask cultivation of *T. reesei* [49]. Recently, the production of SARS-CoV-2 neutralizing nanobodies in *T. reesei* was reported, but the yield was also still 47.4 mg/L [50]. In contrast, in the present study, our inducer-free strain showed the high productivity of nanobodies in the flask cultivation (exceeding 500 mg/L for caplacizumab, Fig. 5cii; exceeding 400 mg/L for 1ZVH and ozoralizumab, Fig. 4bi, iii), indicating that *T. reesei* can be a promising host for small antibody production.

The strategy of fusing CBD-carrier polypeptide and KEX2 linker together with the secretory signal was also effective in nanobody production (Fig. 3). On the other hand, the secretory production of bivalent caplacizumab and trivalent ozoralizumab showed the bands that appeared to be degradation products of His-tagged proteins, which were not seen for the monovalent 1ZVH (Fig. 3c). The bands that are likely the degradation products appeared at approximately the same positions as the monovalent and bivalent nanobodies, presumably suggesting that cleavage was occurring around the flexible linkers to link the V_HH_ fragments (the tri-alanine (AAA) linker for caplacizumab and the Gly-Ser linker (GGGGSGGGGS) for ozoralizumab were used, respectively). The addition of protease inhibitors could significantly reduce these degradants (Fig. 4a) and increase the productivity of the target nanobodies (Fig. 4b). It will be important to design the linker sequences that are not cleaved by proteases while retaining the binding activity and the productivity of nanobodies.

In 1ZVH, the presumable CBD-V_HH_ fusion protein that seemed to be uncleaved at the KEX2 linker was unexpectedly observed in the presence of protease inhibitors (day 2 and 3, Fig. 4ai and Additional file 1: Fig. S3). This result suggests two possibilities that the protease inhibitors may have directly or indirectly acted on the cleavage of the KEX2 linker. For the direct effect, it is required that the protease inhibitors penetrate inside the cells and inhibit the process for cleaving the KEX2 linker, which is generally cleaved in the Golgi apparatus [51]. For the indirect effect, a bit of delayed cell growth, which was conducted by the addition of protease inhibitors (data not shown), might have affected the cleavage efficiency, even though it never changed the total amount of secreted proteins (Additional file 1: Fig. S2). In the future, it will be important to identify proteases that play a crucial role in generating degradation products in nanobodies and delete them by genetic engineering instead of the use of inhibitors. In the production of interferon α-2b (IFNα-2b) using *T. reesei* as a host, the impaired proteases were identified, and high levels of IFNα-2b production were achieved by disrupting the proteases [45]. Additionally, an iterative gene deletion method has been recently developed using an efficient genome editing system in *T. reesei*, successfully constructing the strain with low levels of secretion of the extracellular proteases and the native cellulases/hemicellulases by deleting 11 genes [25]. Together with the deletion of specific harmful protease genes, the additional multiple replacements (deletion) of the endogenous genes of cellulases/hemicellulases as well as *cbh1* and *cbh2* in the inducer-free *T. reesei* strain would offer a promising platform for recombinant protein production.

In biologics produced in eukaryotic cells, glycosylation may often cause problems. The potential consensus sequences (NXS/T) for *N*-linked glycosylation, which should be given special attention due to the large and complicated structure with varied saccharide composition, were not present in the sequences of CBD-linker and nanobodies used in this study (Additional file 2: Table S3). In contrast, because *O*-linked oligosaccharides with relatively simple structures could potentially be modified at any serine or threonine residue, they may have been attached to the nanobodies produced in this study. At the stage of considering production as a biologics rather than diagnostic pharmaceuticals, it will be required to identify the presence or absence of *O*-linked glycosylation and the position of their modification and to examine in detail their impacts on pharmacological effect and antigenicity. In this study, although the analysis of *O*-linked glycosylation has not been performed, we confirmed that at least 1ZVH and caplacizumab produced in *T. reesei* had the binding capacities to each corresponding antigen (Fig. 4d).

In our inducer-free system, two transcriptional activators of cellulase and hemicellulase genes were co-expressed in addition to the deletion of several transcriptional repressors (∆*ace1* and ∆*rce1*) [34]. XYR1, a homologue of *Aspergillus niger* XlnR, is a master regulator for cellulase and hemicellulase genes, and its mutants (XYR1^V821F^ and XYR1^A824V^) have been known to be partially free from the CRE1-dependent CCR in *T. reesei* [52, 53, 54]. However, in the presence of glucose, the expression of mutated XYR1 (XYR1^V821F^ was used in this study) induced strong xylanase expression but had only a limited effect on cellulase induction [34, 53, 54]. Thus, we found that the co-expression of ACE3, which is also known as a positive regulator of cellulases/hemicellulases and has been suggested to form a heterodimer with XYR1, strongly induced cellulase expression in *T. reesei* (partially truncated ACE3 showed even stronger induction) [34]. This is a genus-specific strategy because ACE3 is a transcription factor specific to *Trichoderma* spp. [55]. However, it may be possible to establish a similar strategy for other filamentous fungi with a similar mechanism of cellulase induction.

*Trichoderma* spp. have been used over the past decades to produce enzymes, e.g. as a cocktail of cellulases and hemicellulases on a commercial scale [56]. Although enzyme production for biorefinery still incurs a significant cost, a cost-effective strategy, such as on-site and integrated enzyme manufacturing, is being considered for lignocellulosic biorefinery [56, 57]. In particular, the choice of the primary carbon source accounts for more than 50% of the total cost of enzyme production [34, 58]. Thus, our inducer-free system using glucose as the sole carbon source may contribute to cost-effective recombinant protein production in *T. reesei*. Based on the accumulated industrial knowledge and the utility of enzyme production using *T. reesei*, commercial-scale recombinant protein production using our inducer-free system will be economically feasible in the future, particularly for relatively high value-added proteins.

## Conclusions

In this study, we explored the recently developed inducer-free, cellulase/hemicellulase expression system in *T. reesei* for recombinant protein production. This new platform enabled the utilization of the strongest cellulase promoters for recombinant protein production by replacing the unnecessary cellulase genes and using a common, inexpensive, and soluble sugar, glucose, as the sole carbon source. Thus, we could successfully resolve the challenges and provide unique insights into recombinant protein production in *T. reesei*. Although further study and optimization is warranted, our approach has great potential as a new alternative method for high-level recombinant protein production in other non-conventional microorganisms.

## Methods

### Strains and media

*T. reesei* strains used in this study are listed in Additional file 2: Table S1. Strains were maintained on potato dextrose agar (PDA; Difco Laboratories, Detroit, MI, USA) plates. The basal medium comprised 0.14% (w/v) (NH_4_)_2_SO_4_, 0.2% (w/v) KH_2_PO_4_, 0.03% (w/v) CaCl_2_·2H_2_O, 0.03% (w/v) MgSO_4_ 7H_2_O, 0.1% (w/v) polypeptone, 0.05% (w/v) yeast extract, 0.1% (w/v) Tween 80, and 0.1% (w/v) trace element solution in 50 mM Na-tartrate buffer (pH 4.0). The trace element solution contained 6 mg H_3_BO_3_, 26 mg (NH_4_)_6_Mo_7_O_24_·4H_2_O, 100 mg FeCl_3_·6H_2_O, 40 mg CuSO_4_·5H_2_O, 8 mg MnCl_2_·4H_2_O, and 200 mg ZnCl_2_ in 100 mL of distilled water.

### Shake flask cultivation

For preculture enzyme production, 4 × 10^5^ spores of each strain were inoculated into 2 mL of basal medium [52] containing 1% (w/v) glucose in a 10-mL culture tube. Spores were counted using a Thoma hemocytometer (Sunlead Glass Corp., Saitama, Japan). Preculturing was carried out by shaking at 220 rpm, at 28 °C for 2 days. For each of the main cultures, 500 µL of the preculture was inoculated into 50 mL of basal medium containing 3% (w/v) powdered cellulose (KC FLOCK W-400G, Nippon Paper Industries, Tokyo, Japan) or 3% (w/v) glucose, and 1.28% (w/v) diammonium hydrogen citrate in a 500 mL flask. For the protease inhibition study, one tablet of cOmplete, EDTA-free Protease Inhibitor Cocktail (Roche Diagnostics, Basel, Switzerland) was added per 50 mL of medium before the start of culture. The main culture was shaken at 220 rpm, at 28 °C for up to 4 days. For sampling, cells were removed from the culture broth by centrifugation at 16,000*g* for 5 min, and the supernatant was filtered through a 0.20 μm cellulose acetate membrane filter (13CP020AN; Advantec, Tokyo, Japan). All experiments were carried out in triplicate.

### Molecular cloning and construction of the expression cassettes

The genes of interest were amplified from the genomic DNA of *T. reesei* strain PC-3-7, and a vector fragment was amplified by inverse polymerase chain reaction (PCR) using pUC118 (Takara Bio, Shiga, Japan) as the template. The amplified fragments, which have been pre-designed to add a SwaI cleavage site using primers, were ligated using an In-Fusion HD Cloning Kit (Clontech Laboratories, Mountain View, CA, USA) according to the manufacturer’s protocol. *Escherichia coli* DH5a was used as a cloning host, and a NucleoSpin^®^ Plasmid miniprep kit (Takara Bio) was used to purify the plasmid DNA. More details on the cloned gene and primers are provided in an Additional file 2: Table S2. The nanobody expression cassettes were constructed by the fragment of artificial gene synthesis (Additional file 2: Table S4) procured from the amino acid sequence of a nanobody with CBD-carrier polypeptide and KEX2 linker (Fig. 3a, SCK) shown in Additional file 2: Table S2 and the PCR fragment with the primer pair shown in Additional file 2: Table S5. The expression cassettes of secretory signal peptide (Fig. 3a, S) and CBD-carrier polypeptide (Fig. 3a, SC) addition were obtained by inverse PCR from expression cassettes of CSK patterns (pUC-V014, pUC-V017, pUC-V020) as templates.

### Transformation of *T. reesei*

Before transformation of *T. reesei*, the plasmid was linearized with SwaI and transformed with a modified protoplast-polyethylene glycol (PEG) method [60], in which 20 mg/mL of Yatalase (Takara Bio) was used as the protoplasting enzyme instead of Novozyme 234 (Novozymes, Bagsværd, Denmark). The transformed protoplasts were plated on minimal transformation medium [2.0% (w/v) glucose, 18.27% (w/v) sorbitol, 0.5% (w/v) (NH_4_)_2_SO_4_, 0.2% (w/v) CaCl_2_, 0.06% (w/v) MgSO_4_, 0.21% (w/v) CsCl, and 0.1% (w/v) trace element solution in 100 mM KH_2_PO_4_ buffer (pH 5.5)] for the *pyr4* marker. The trace element solution contained 500 mg FeSO_4_·7H_2_O, 200 mg CoCl_2_, 160 mg MnSO_4_·H_2_O, and 140 mg ZnSO_4_·7H_2_O in 100 mL of distilled water. After 2 weeks of incubation at 30 °C, candidate transformants were streaked twice on selective plates (each minimal transformation medium without sorbitol) for several days at 30 °C for single-colony isolation. Single colonies were then transferred to PDA plates for 1 week at 30 °C to allow for the formation of conidia. One transformant was confirmed by colony PCR using KOD One (Toyobo, Osaka, Japan), according to the manufacturer’s protocol. To transform the resulting transformants again using a PDA medium containing 0.2% (w/v) 5-fluoroorotic acid (5-FOA) monohydrate, a strain that acquired 5-FOA resistance again (*pyr4* pop-out through homologous recombination) was selected.

### Protein concentration analysis with BCA assay

The protein concentrations were measured with a Pierce BCA Protein Assay Kit (Thermo Fisher Scientific, Waltham, MA, USA) with bovine serum albumin as the standard, according to the manufacturer’s microplate protocol. The absorbance of all samples and standards was measured at 562 nm using a microplate reader (Molecular Devices, San Jose, CA, USA).

### Protein composition analysis

SDS-PAGE was carried out using Any kD Mini-PROTEAN TGX Precast Protein Gels (Bio-Rad Laboratories, Hercules, CA, USA) for 30 min at 200 V. The gel was activated for 5 min and imaged using the ChemiDoc MP imaging system (Bio-Rad Laboratories). Precision Plus Protein Unstained Standard (5 µL; Bio-Rad Laboratories) was used as a molecular mass marker. The molecular weight of the protein bands was estimated using Image Lab software (Bio-Rad Laboratories), and the protein bands were annotated using the positions corresponding to previously reported cellulases and hemicellulases [35].

### Western blot analysis

After SDS-PAGE, proteins were transferred to PVDF membranes using Trans-Blot Turbo Mini PVDF Transfer Packs (Bio-Rad Laboratories) and Trans-Blot Turbo System (Bio-Rad). HRP-conjugated Anti-6x-His Tag Monoclonal Antibody (3D5) (Thermo Fisher Scientific) was bound using the iBind Western System (Invitrogen, Waltham, MA, USA), and His-tagged protein was detected with 1-Step Ultra TMB-Blotting Solution (Thermo Fisher Scientific).

### His-tagged protein purification

A 1.5 mL of culture supernatant adjusted to pH 9.0, 150 µL of Ni-NTA Agarose (FUJIFILM Wako Chemicals, Osaka, Japan) was added and mixed in a rotator at 4 °C, for 30 min. After centrifugation at 500g for 5 min, the supernatant was removed and 500 µL of Wash Buffer (20 mM imidazole, 500 mM NaCl, 20 mM Tris-HCl pH 8.0) was added. After suspension by vortexing, centrifugation was performed at 500g for 5 min and the supernatant was removed. After repeating this washing twice, 50 µL of Elution Buffer (300 mM imidazole, 500 mM NaCl, 20 mM Tris-HCl pH 8.0) was added, and mixed in a rotator at 4 °C, for 30 min. After centrifugation at 500g for 5 min, the supernatant was transferred to a new tube, and His-tagged protein was obtained.

### Determination of V_HH_ antibody (nanobody) concentration in culture supernatant

The concentration of nanobody in the culture supernatant was estimated by western blotting using appropriately diluted culture supernatants. As a standard, the concentration of each His-tagged purified nanobody was quantified by BCA assay (see above), and the quantification was performed within the range of linearity between the band intensity and nanobody concentration.

### Kinetics analysis of nanobody and antigens

The BLItz System (Fortebio, Fremont CA, USA) with Bio-Layer Interferometry was used for kinetics analysis. Anti-Penta-HIS (HIS1K) was selected as the sensor and hydrophilized by immersion in PBS-T (phosphate-buffered saline with 0.1% Tween-20) for 10 min. The nanobody was then diluted to 100 mg/L and a dilution series of antigens (for 1ZVH: lysozyme from chicken egg white, L4919, Sigma-Aldrich, St. Louis, MO, USA; for caplacizumab: Human von Willebrand Factor (vWF), HCVWF-0191, Haematologic Technologies, Essex, VT, USA) was prepared. Measurements were performed using methods of Initiation (Sample diluent buffer, 60 s), Loading (nanobody, 90 s), Baseline (Sample diluent buffer, 60 s), Association (Antigen, 180 s), and Dissociation (Sample diluent buffer, 300 s). The sensorgram without antigen was used as a reference, corrected for background, and the sensorgram was subjected to global fitting to calculate the *K*_D_ value.

### Statistical analysis

All experiments were performed with at least three independent samples. Error bars indicate the standard deviation (SD) of the mean of triplicates. Statistical significance was determined by the two-tailed unpaired Student’s *t*-test. Within each set of experiments, *p* < 0.05 was considered significant.

## Supplementary Information

**Additional file 1**: **Figure S1**. Total secreted proteins under single replacement of *cbh1* with three expression patterns of nanobody genes. *T. reesei* E1AB1-XA3 strain and transformants were cultivated in shake flasks on a non-inducing medium containing 3% glucose. Total secreted proteins after 4 days of cultivation. **Figure S2**. Total secreted protein under conditions with/without protease inhibitors. *T. reesei* E1AB1-XA3 strain-based transformants were cultivated in shake flasks on a non-inducing medium containing 3% glucose and with/without protease inhibitor cocktail. Total secreted proteins after 4 days of cultivation. **Figure S3**. Adsorption of 1ZVH-CBD fusion protein via cellulose treatment. His-tagged proteins were purified from day 2 supernatant of 1ZVH expressed in SC and SCK patterns using Ni-NTA beads. Centrifuged  with adsorption on cellulose, and untreated purified samples were subjected to SDS-PAGE. Open arrowhead indicates the adsorbed protein, corresponding to the 1ZVH-CBD fusion protein.  **Figure S4**. Western blotting of culture supernatants of *cbh1* and *cbh2* double replacement strains. Using *T. reesei* E1AB1 and E1AB1-XA3 strains as parental strains, single replacement of *cbh1* and double replacement of *cbh1* and *cbh2* with caplasizumab expression cassette that applied CBD-carrier polypeptide and KEX2 linker pattern were performed. E1AB1 strain and transformants were cultivated in shake flasks on an inducing medium containing 3% cellulose, and *T. reesei* E1AB1-XA3 strain-based transformants were cultivated in shake flasks on a non-inducing medium containing 3% glucose. Western blotting analysis using anti-His tag antibody on gels were performed on SDS-PAGE loaded with 5 μL of8-fold and16-fold diluted supernatant.

**Additional file 2: Table S1.** *T. reesei* strains used in this study. **Table S2.** Primer pairs for gene cloning. **Table S3.** Amino acid sequence of nanobodies. **Table S4.** Nucleotide sequences of artificial gene synthesis. **Table S5.** Constructed expression cassettes and primer pairs.

## Data Availability

The protein and nucleotide sequences of *T. reesei* used in this study can be referenced from the JGI genome database (https://mycocosm.jgi.doe.gov/pages/search-for-genes.jsf?organism=Trire2) under the following accession IDs: CBH1_tre123989, CBH2_tre 72,567, PEP1_ tre74156, GLA1_ tre1885. For nanobody, PDB ID: 1ZVH as anti-lysozyme V_HH_, and the following are available as DrugBank Accession number: caplacizumab_DB06081, ozoralizumab_DB12014.

## Abbreviations

CAZy: Carbohydrate active enzyme
CBD: Cellulose-binding domain
CBH: Cellobiohydrolase
CCR: Carbon catabolite repression
POI: Protein of interest
SDS-PAGE: Sodium dodecyl sulfate polyacrylamide gel electrophoresis
V_HH_: Variable domain of heavy chain of heavy chain antibody
