# Supplementary material for: Inducer-free recombinant protein production in Trichoderma reesei: secretory production of endogenous enzymes and heterologous nanobodies using glucose as the sole carbon source
Source: Microb Cell Fact. 2023 May 19;22:103. doi: 10.1186/s12934-023-02109-y (PMC10197057; doi:10.1186/s12934-023-02109-y)
Supplement: Supplementary file 1 — Additional file 1: Figure S1. Total secreted proteins under single replacement of cbh1 with three expression patterns of nanobody genes. T. reesei E1AB1-XA3 strain and transformants were cultivated in shake flasks on a non-inducing medium containing 3% glucose. Total secreted proteins after 4 days of cultivation. Figure S2. Total secreted protein under conditions with/without protease inhibitors. T. reesei E1AB1-XA3 strain-based transformants were cultivated in shake flasks on a non-inducing medium containing 3% glucose and with/without protease inhibitor cocktail. Total secreted proteins after 4 days of cultivation. Figure S3. Adsorption of 1ZVH-CBD fusion protein via cellulose treatment. His-tagged proteins were purified from day 2 supernatant of 1ZVH expressed in SC and SCK patterns using Ni-NTA beads. Centrifuged with adsorption on cellulose, and untreated purified samples were subjected to SDS-PAGE. Open arrowhead indicates the adsorbed protein, corresponding to the 1ZVH-CBD fusion protein. Figure S4. Western blotting of culture supernatants of cbh1 and cbh2 double replacement strains. Using T. reesei E1AB1 and E1AB1-XA3 strains as parental strains, single replacement of cbh1 and double replacement of cbh1 and cbh2 with caplasizumab expression cassette that applied CBD-carrier polypeptide and KEX2 linker pattern were performed. E1AB1 strain and transformants were cultivated in shake flasks on an inducing medium containing 3% cellulose, and T. reesei E1AB1-XA3 strain-based transformants were cultivated in shake flasks on a non-inducing medium containing 3% glucose. Western blotting analysis using anti-His tag antibody on gels were performed on SDS-PAGE loaded with 5 μL of8-fold and16-fold diluted supernatant. [file 12934_2023_2109_MOESM1_ESM.pdf]

Additional file 1 (Supplementary Figures)

Inducer-free recombinant protein production in *Trichoderma reesei*: Secretory production of endogenous enzymes and heterologous nanobodies using glucose as the sole carbon source

Toshiharu Arai[1,2*], Mayumi Wada[1], Hiroki Nishiguchi[1], Yasushi Takimura[1], Jun Ishii[2,3*]

1. Biological Science Research, Kao Corporation, 1334 Minato, Wakayama, Wakayama 640-8580, Japan
2. Graduate School of Science, Technology and Innovation, Kobe University, 1-1 Rokkodai, Nada, Kobe, 657-8501, Japan
3. Engineering Biology Research Center, Kobe University, 1-1 Rokkodai, Nada, Kobe, 657-8501, Japan

*Correspondence: arai.toshiharu@kao.com
junjun@port.kobe-u.ac.jp

Figure S1

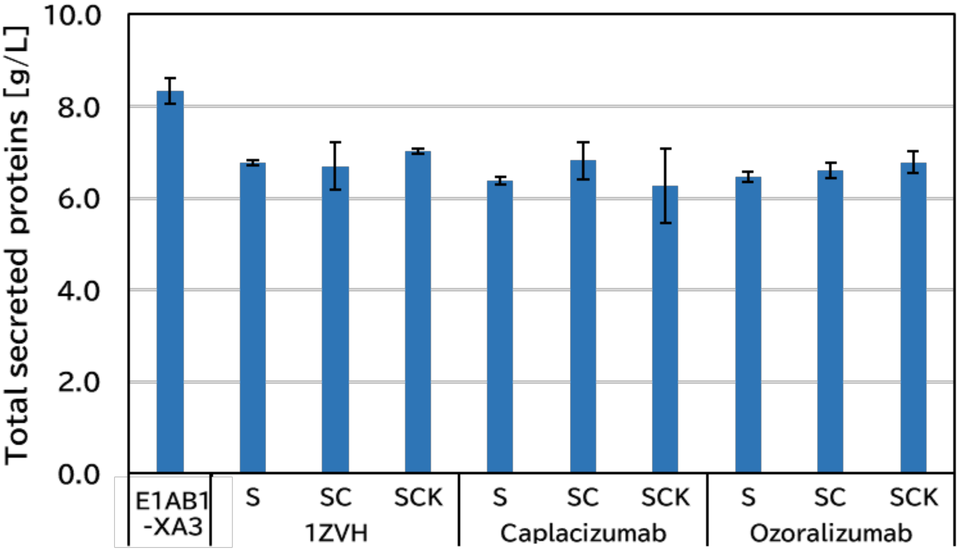

Fig. S1 Total secreted proteins under single replacement of *cbh1* with three expression patterns of nanobody genes

T. reesei E1AB1-XA3 strain and transformants were cultivated in shake flasks on a non-inducing medium containing 3% glucose. Total secreted proteins after 4 days of cultivation.

Figure S2

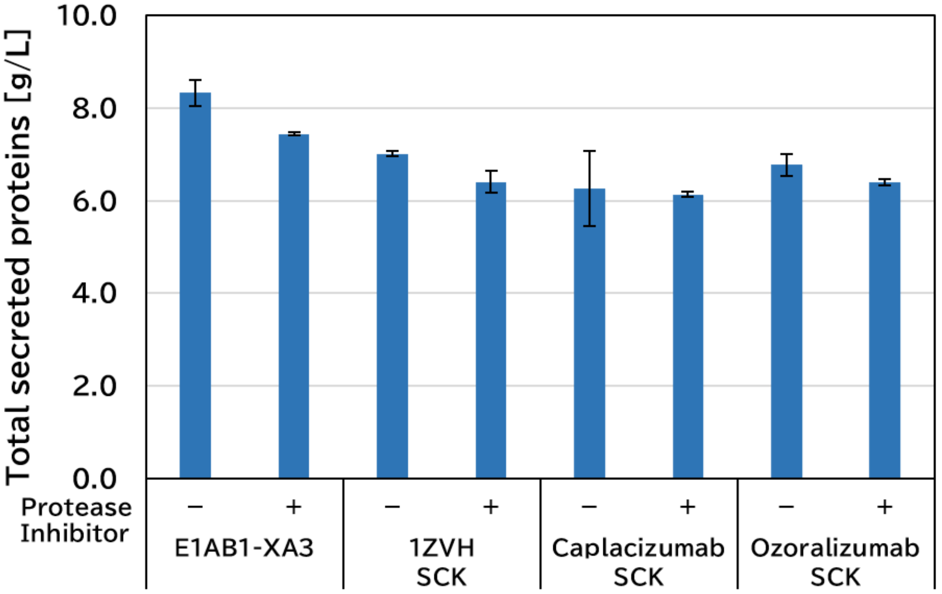

Fig. S2 Total secreted protein under conditions with (+)/without (-) protease inhibitors

T. reesei E1AB1-XA3 strain-based transformants were cultivated in shake flasks on a non-inducing medium containing 3% glucose and with (+)/without (-) protease inhibitor cocktail. Total secreted proteins after 4 days of cultivation.

Figure S3

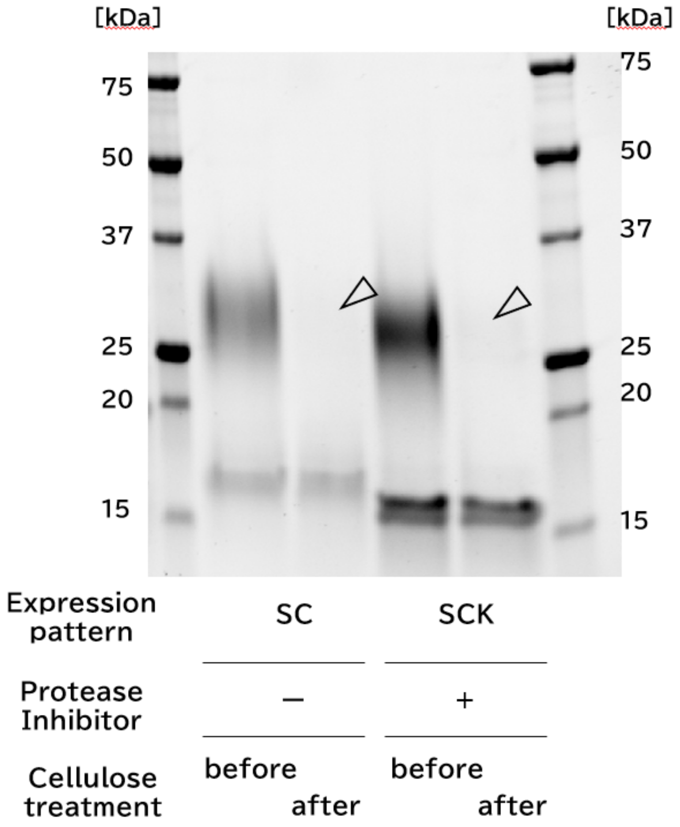

Fig. S3 Adsorption of 1ZVH-CBD fusion protein via cellulose treatment

His-tagged proteins were purified from day 2 supernatant of 1ZVH expressed in SC (without protease inhibitor) and SCK (with protease inhibitor) patterns using Ni-NTA beads. Centrifuged supernatants treated with adsorption on cellulose (10 wt% microcrystalline cellulose added, mixed, and waited at room temperature for 10 minutes), and untreated purified samples were subjected to SDS-PAGE. Open arrowhead indicates the adsorbed protein, corresponding to the 1ZVH-CBD fusion protein.

Figure S4

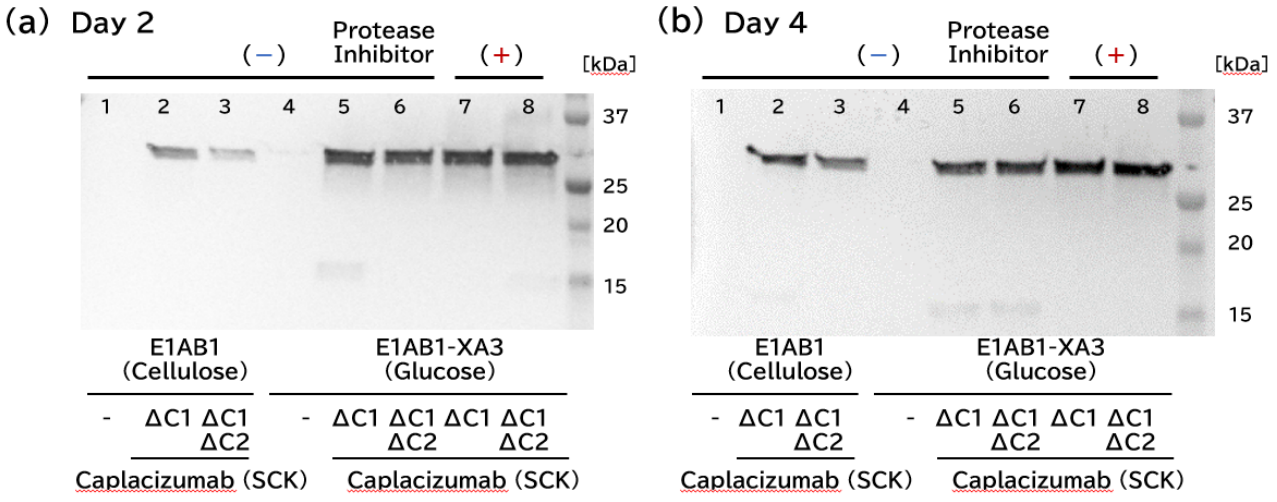

Fig. S4 Western blotting of culture supernatants of *cbh1* and *cbh2* double replacement strains

Using *T. reesei* E1AB1 and E1AB1-XA3 strains as parental strains, single replacement of *cbh1* and double replacement of *cbh1* and *cbh2* with caplasizumab expression cassette that applied CBD-carrier polypeptide and KEX2 linker (SCK) pattern were performed. E1AB1 strain and transformants were cultivated in shake flasks on an inducing medium containing 3% cellulose, and *T. reesei* E1AB1-XA3 strain-based transformants were cultivated in shake flasks on a non-inducing medium containing 3% glucose. Western blotting analysis using anti-His tag antibody on gels were performed on SDS-PAGE loaded with 5 µL of (a) 8-fold and (b) 16-fold diluted supernatant.
