# Supplementary material for: Inducer-free recombinant protein production in Trichoderma reesei: secretory production of endogenous enzymes and heterologous nanobodies using glucose as the sole carbon source
Source: Microb Cell Fact. 2023 May 19;22:103. doi: 10.1186/s12934-023-02109-y (PMC10197057; doi:10.1186/s12934-023-02109-y)
Supplement: Supplementary file 2 — Additional file 2: Table S1. T. reesei strains used in this study. Table S2. Primer pairs for gene cloning. Table S3. Amino acid sequence of nanobodies. Table S4. Nucleotide sequences of artificial gene synthesis. Table S5. Constructed expression cassettes and primer pairs. [file 12934_2023_2109_MOESM2_ESM.pdf]

Additional file 2 (Supplementary Tables)

Inducer-free recombinant protein production in *Trichoderma reesei*: Secretory production of endogenous enzymes and heterologous nanobodies using glucose as the sole carbon source

Toshiharu Arai[1,2*], Mayumi Wada[1], Hiroki Nishiguchi[1], Yasushi Takimura[1], Jun Ishii[2,3*]

1. Biological Science Research, Kao Corporation, 1334 Minato, Wakayama, Wakayama 640-8580, Japan
2. Graduate School of Science, Technology and Innovation, Kobe University, 1-1 Rokkodai, Nada, Kobe, 657-8501, Japan
3. Engineering Biology Research Center, Kobe University, 1-1 Rokkodai, Nada, Kobe, 657-8501, Japan

*Correspondence: arai.toshiharu@kao.com
junjun@port.kobe-u.ac.jp

Table S1 *T. reesei* strains used in this study

Strains	Description	Genotypes
PC-3-7 (ATCC 65589)	Need for induction, Hyper-secreting mutant	-
E1AB1	Need for induction, More released from CCR	*amdS*+, Δ*tubB*::*egl1*p-*aabgl1*
E1AB1-XA3	Inducer-free	E1AB1, Δ*ace1*::*act1*p-*xyr1*(V821F), Δ*rce1*::*act1*p-*ace3*
E1AB1-XA3ΔC1-Pep1	Inducer-free	E1AB1-XA3, Δ*cbh1*::*cbh1*p-*pep1*
E1AB1-XA3ΔC1-Gla1	Inducer-free	E1AB1-XA3, Δ*cbh1*::*cbh1*p-*gla1*
E1AB1-XA3ΔC1-1ZVH-S	Inducer-free	E1AB1-XA3, Δ*cbh1*::*cbh1*p-*1zvh* (*cbh2* SS)
E1AB1-XA3ΔC1-1ZVH-SC	Inducer-free	E1AB1-XA3, Δ*cbh1*::*cbh1*p-*1zvh* (*cbh2* SS+CBD)
E1AB1-XA3ΔC1-1ZVH-SCK	Inducer-free	E1AB1-XA3, Δ*cbh1*::*cbh1*p-*1zvh* (*cbh2* SS+CBD+Kex2 linker)
E1AB1-XA3ΔC1-Cap-S	Inducer-free	E1AB1-XA3, Δ*cbh1*::*cbh1*p-*caplacizumab* (*cbh2* SS)
E1AB1-XA3ΔC1-Cap-SC	Inducer-free	E1AB1-XA3, Δ*cbh1*::*cbh1*p-*caplacizumab* (*cbh2* SS+CBD)
E1AB1-XA3ΔC1-Cap-SCK	Inducer-free	E1AB1-XA3, Δ*cbh1*::*cbh1*p-*caplacizumab* (*cbh2* SS+CBD+Kex2 linker)
E1AB1-XA3ΔC1-Ozo-S	Inducer-free	E1AB1-XA3, Δ*cbh1*::*cbh1*p-*ozoralizumab* (*cbh2* SS)
E1AB1-XA3ΔC1-Ozo-SC	Inducer-free	E1AB1-XA3, Δ*cbh1*::*cbh1*p-*ozoralizumab* (*cbh2* SS+CBD)
E1AB1-XA3ΔC1-Ozo-SCK	Inducer-free	E1AB1-XA3, Δ*cbh1*::*cbh1*p-*ozoralizumab* (*cbh2* SS+CBD+Kex2 linker)
E1AB1-XA3ΔC1ΔC2-Cap-SCK	Inducer-free	E1AB1-XA3ΔC1-Cap-SCK, Δ*cbh2*::*cbh2*p-caplacizumab (*cbh2* SS+CBD+Kex2 linker)
E1AB1ΔC1-Cap-SCK	Need for induction	E1AB1, Δ*cbh1*::*cbh1*p-*caplacizumab* (*cbh2* SS+CBD+Kex2 linker)
E1AB1ΔC1ΔC2-Cap-SCK	Need for induction	E1AB1ΔC1-Cap-SCK, Δ*cbh2*::*cbh2*p-caplacizumab (*cbh2* SS+CBD+Kex2 linker)

Table S2 Primer pairs for gene cloning

Plasmid		fragment	Forward Primer	Reverse Primer	Templete
pUC-V001	pUC-118		-	-	-
pUC-V002	pUC-Tcbh1	1	CTAGAGTATTTAAATAGCTCCGTGGCGAAAGCCTG	TGCAGGTATTTAAATGGAGCCATACTGGCGGGAAC	Genome DNA
		2	ATTTAAATACCTGCAGGCATGCAAGCTT	ATTTAAATACTCTAGAGGATCCCCGGGT	pUC-V001
pUC-V003	pUC-pyr4	1	CTAGAGTATTTAAATCAAACCAGCCAAGGTAGGTA	TGCAGGTATTTAAATATCACATGTCAATGTCACAA	Genome DNA
		2	ATTTAAATACCTGCAGGCATGCAAGCTT	ATTTAAATACTCTAGAGGATCCCCGGGT	pUC-V001
pUC-K004	pUC-Tr120830	1	CTAGAGTATTTAAATTTTCGCCTTAGCGGTTCCAT	TGCAGGTATTTAAATTTAATACTCGGCAGTCTCGTCAG	Genome DNA
		2	ATTTAAATACCTGCAGGCATGCAAGCTT	ATTTAAATACTCTAGAGGATCCCCGGGT	pUC-V001
pUC-V005	pUC-Tcbh1-Tr120830-3'	1	CTAGAGTATTTAAATAGCTCCGTGGCGAAAGCCTG	AAGCGCGCGGTACTTCTCGGCTACGTTGTCATCGT	pUC-V002
		2	AAGTACCGCGCGCTTGACAA	TGCAGGTATTTAAATCTGAATGCCCGGTGGTAAGC	pUC-V004
pUC-V006	pUC-pyr4-Tr120830-3'	1	CTAGAGTATTTAAATCAAACCAGCCAAGGTAGGTA	CCATCACATGTCAATGTCAC	pUC-V003
		2	ATTGACATGTGATGGAAGTACCGCGCGCTTGACAA	TGCAGGTATTTAAATTTGGTTCTTGGTTTGGAGGG	pUC-V004
pUC-V007	pUC-Tcbh1-Tr120830-3'-pyr4-Tr120830-3' (pyr4*)	1	CTAGAGTATTTAAATAGCTCCGTGGCGAAAGCCTG	CTGAATGCCCGGTGGTAAGC	pUC-V005
		2	CCACCGGGCATTCAGCAAACCAGCCAAGGTAGGTA	TGCAGGTATTTAAATTTGGTTCTTGGTTTGGAGGG	pUC-V006
pUC-V008	pUC-cbh1	1	CTAGAGTATTTAAATACTCGTATTCGCCCTAAACC	TGCAGGTATTTAAATCAACACAGTTTCAGCCCTAG	Genome DNA
		2	ATTTAAATACCTGCAGGCATGCAAGCTT	ATTTAAATACTCTAGAGGATCCCCGGGT	pUC-V001
pUC-V009	pUC-cbh2	1	CTAGAGTATTTAAATCTCCCAGAAGTTACCCAACC	TGCAGGTATTTAAATGAAGGACCAAAGAACCTCGC	Genome DNA
		2	ATTTAAATACCTGCAGGCATGCAAGCTT	ATTTAAATACTCTAGAGGATCCCCGGGT	pUC-V001
pUC-V010	pUC-pep1	1	CTAGAGTATTTAAATATTGGGCCATACGAGGCCTG	TGCAGGTATTTAAATCATAGCATGAGACGCTTTCC	Genome DNA
		2	ATTTAAATACCTGCAGGCATGCAAGCTT	ATTTAAATACTCTAGAGGATCCCCGGGT	pUC-V001
pUC-V011	pUC-gla1	1	CTAGAGTATTTAAATATCCTGCTCTTCCAGCCCAC	TGCAGGTATTTAAATAAGCGCTCTCATTGTCCAAC	Genome DNA
		2	ATTTAAATACCTGCAGGCATGCAAGCTT	ATTTAAATACTCTAGAGGATCCCCGGGT	pUC-V001

Table S3 Amino acid sequence of nanobodies

CBH2 secretion signal sequence

CBH2 cellulose binding domain+linker

KEX2 cleavage sites

V_{HH} sequence

Linkers in V_{HH}

6×His

**1ZVH**	**S**	MIVGILTTLATLATLAASVPDVQLVESGGGSVQAGGSLRLSCAASGYIASINYLGWFRQAPGKEREGVAAVSPAGGTPYYADSVKGRFTVSLDNAENTVYLQMNSLKPEDTALYYCAAARQGWYIPLNSYGYNYWGQGTQVTVSSRGRHHHHHH*
	**SC**	MIVGILTTLATLATLAASVPLEERQACSSVWGQCGGQNWSGPTCCASGSTCVYSNDYYSQCLPGAASSSSSTRAASTTSRVSPTTSDVQLVESGGGSVQAGGSLRLSCAASGYIASINYLGWFRQAPGKEREGVAAVSPAGGTPYYADSVKGRFTVSLDNAENTVYLQMNSLKPEDTALYYCAAARQGWYIPLNSYGYNYWGQGTQVTVSSRGRHHHHHH*
	**SCK**	MIVGILTTLATLATLAASVPLEERQACSSVWGQCGGQNWSGPTCCASGSTCVYSNDYYSQCLPGAASSSSSTRAASTTSRVSPTTSRDKRDVQLVESGGGSVQAGGSLRLSCAASGYIASINYLGWFRQAPGKEREGVAAVSPAGGTPYYADSVKGRFTVSLDNAENTVYLQMNSLKPEDTALYYCAAARQGWYIPLNSYGYNYWGQGTQVTVSSRGRHHHHHH*
**Caplacizumab**	**S**	MIVGILTTLATLATLAASVPEVQLVESGGGLVQPGGSLRLSCAASGRTFSYNPMGWFRQAPGKGRELVAAISRTGGSTYYPDSVEGRFTISRDNAKRMVYLQMNSLRAEDTAVYYCAAAGVRAEDGRVRTLPSEYTFWGQGTQVTVSSAAAEVQLVESGGGLVQPGGSLRLSCAASGRTFSYNPMGWFRQAPGKGRELVAAISRTGGSTYYPDSVEGRFTISRDNAKRMVYLQMNSLRAEDTAVYYCAAAGVRAEDGRVRTLPSEYTFWGQGTQVTVSSHHHHHH*
	**SC**	MIVGILTTLATLATLAASVPLEERQACSSVWGQCGGQNWSGPTCCASGSTCVYSNDYYSQCLPGAASSSSSTRAASTTSRVSPTTSEVQLVESGGGLVQPGGSLRLSCAASGRTFSYNPMGWFRQAPGKGRELVAAISRTGGSTYYPDSVEGRFTISRDNAKRMVYLQMNSLRAEDTAVYYCAAAGVRAEDGRVRTLPSEYTFWGQGTQVTVSSAAAEVQLVESGGGLVQPGGSLRLSCAASGRTFSYNPMGWFRQAPGKGRELVAAISRTGGSTYYPDSVEGRFTISRDNAKRMVYLQMNSLRAEDTAVYYCAAAGVRAEDGRVRTLPSEYTFWGQGTQVTVSSHHHHHH*
	**SCK**	MIVGILTTLATLATLAASVPLEERQACSSVWGQCGGQNWSGPTCCASGSTCVYSNDYYSQCLPGAASSSSSTRAASTTSRVSPTTSRDKREVQLVESGGGLVQPGGSLRLSCAASGRTFSYNPMGWFRQAPGKGRELVAAISRTGGSTYYPDSVEGRFTISRDNAKRMVYLQMNSLRAEDTAVYYCAAAGVRAEDGRVRTLPSEYTFWGQGTQVTVSSAAAEVQLVESGGGLVQPGGSLRLSCAASGRTFSYNPMGWFRQAPGKGRELVAAISRTGGSTYYPDSVEGRFTISRDNAKRMVYLQMNSLRAEDTAVYYCAAAGVRAEDGRVRTLPSEYTFWGQGTQVTVSSHHHHHH*
**Ozoralizumab**	**S**	MIVGILTTLATLATLAASVPEVQLVESGGGLVQPGGSLRLSCAASGFTFSDYWMYWVRQAPGKGLEWVSEINTNGLITKYPDSVKGRFTISRDNAKNTLYLQMNSLRPEDTAVYYCARSPSGFNRGQGTLVTVSSGGGGSGGGSEVQLVESGGGLVQPGNSLRLSCAASGFTFSSFGMSWVRQAPGKGLEWVSSISGSGSDTLYADSVKGRFTISRDNAKTTLYLQMNSLRPEDTAVYYCTIGGSLSRSSQGTLVTVSSGGGGSGGGSEVQLVESGGGLVQPGGSLRLSCAASGFTFSDYWMYWVRQAPGKGLEWVSEINTNGLITKYPDSVKGRFTISRDNAKNTLYLQMNSLRPEDTAVYYCARSPSGFNRGQGTLVTVSSHHHHHH*
	**SC**	MIVGILTTLATLATLAASVPLEERQACSSVWGQCGGQNWSGPTCCASGSTCVYSNDYYSQCLPGAASSSSSTRAASTTSRVSPTTSEVQLVESGGGLVQPGGSLRLSCAASGFTFSDYWMYWVRQAPGKGLEWVSEINTNGLITKYPDSVKGRFTISRDNAKNTLYLQMNSLRPEDTAVYYCARSPSGFNRGQGTLVTVSSGGGGSGGGSEVQLVESGGGLVQPGNSLRLSCAASGFTFSSFGMSWVRQAPGKGLEWVSSISGSGSDTLYADSVKGRFTISRDNAKTTLYLQMNSLRPEDTAVYYCTIGGSLSRSSQGTLVTVSSGGGGSGGGSEVQLVESGGGLVQPGGSLRLSCAASGFTFSDYWMYWVRQAPGKGLEWVSEINTNGLITKYPDSVKGRFTISRDNAKNTLYLQMNSLRPEDTAVYYCARSPSGFNRGQGTLVTVSSHHHHHH*
	**SCK**	MIVGILTTLATLATLAASVPLEERQACSSVWGQCGGQNWSGPTCCASGSTCVYSNDYYSQCLPGAASSSSSTRAASTTSRVSPTTSRDKREVQLVESGGGLVQPGGSLRLSCAASGFTFSDYWMYWVRQAPGKGLEWVSEINTNGLITKYPDSVKGRFTISRDNAKNTLYLQMNSLRPEDTAVYYCARSPSGFNRGQGTLVTVSSGGGGSGGGSEVQLVESGGGLVQPGNSLRLSCAASGFTFSSFGMSWVRQAPGKGLEWVSSISGSGSDTLYADSVKGRFTISRDNAKTTLYLQMNSLRPEDTAVYYCTIGGSLSRSSQGTLVTVSSGGGGSGGGSEVQLVESGGGLVQPGGSLRLSCAASGFTFSDYWMYWVRQAPGKGLEWVSEINTNGLITKYPDSVKGRFTISRDNAKNTLYLQMNSLRPEDTAVYYCARSPSGFNRGQGTLVTVSSHHHHHH*

Table S4 Nucleotide sequences of artificial gene synthesis

**1ZVH (AGS-1)**	**SCK**	ATGATCGTCGGCATTCTCACCACGCTCGCTACGCTCGCTACCCTCGCTGCTAGCGTCCCTCTGGAGGAGCGCCAAGCTTGCTCCTCCGTCTGGGGTCAATGCGGTGGCC<br>AGAACTGGTCCGGTCCCACGTGCTGCGCCAGCGGTAGCACGTGCGTCTACTCCAACGACTACTACAGCCAGTGCCTCCCCGGCGCCGCTAGCTCCAGCAGCTCCACC<br>CGAGCTGCTTCCACCACCTCCCGAGTCAGCCCTACCACCAGCCGAGACAAGCGAGATGTCCAGCTCGTCGAAAGCGGCGGTGGCAGCGTCCAAGCTGGTGGTTCGC<br>TCCGACTGAGCTGCGCCGCCAGCGGTTACATCGCCAGCATCAACTACCTCGGCTGGTTCCGCCAAGCCCCCGGCAAGGAGCGAGAGGGTGTCGCTGCTGTCTCCCCC<br>GCCGGTGGTACCCCCTACTATGCCGACAGCGTGAAGGGCCGCTTCACCGTCAGCCTCGACAATGCCGAGAACACGGTCTATCTCCAGATGAACTCGCTGAAGCCCGA<br>GGACACCGCTCTGTACTACTGCGCTGCCGCCCGACAAGGCTGGTACATCCCCCTCAACAGCTACGGCTACAACTACTGGGGCCAAGGCACCCAAGTCACCGTCTCCA<br>GCCGAGGCCGCCACCATCACCACCATCACTAA
**Caplacizumab (AGS-2)**	**SCK**	ATGATCGTCGGCATCCTCACGACGCTCGCTACCCTCGCTACGCTCGCTGCTTCCGTCCCTCTCGAGGAGCGCCAAGCCTGTAGCAGCGTCTGGGGCCAGTGTGGCGGC<br>CAAAACTGGAGCGGCCCTACCTGCTGTGCTTCCGGCAGCACGTGCGTCTACTCCAACGACTACTACTCCCAATGCCTCCCCGGTGCTGCTAGCTCCAGCAGCAGCACG<br>CGAGCTGCTAGCACGACCTCCCGAGTGAGCCCTACCACGTCCCGCGACAAGCGAGAGGTCCAACTGGTGGAGTCCGGTGGCGGCCTCGTCCAACCTGGTGGCTCCCT<br>CCGACTGTCCTGCGCTGCTAGCGGTCGCACCTTCTCCTACAACCCCATGGGCTGGTTTCGACAGGCTCCTGGCAAAGGCCGCGAGCTGGTCGCTGCCATTTCCCGAAC<br>GGGCGGCTCCACCTACTACCCCGATTCCGTCGAGGGCCGATTTACCATCAGCCGAGACAACGCCAAGCGAATGGTCTACCTGCAGATGAACAGCCTCCGAGCTGAGG<br>ACACGGCTGTGTACTACTGTGCTGCTGCCGGTGTCCGAGCCGAAGATGGTCGAGTCCGCACCCTGCCCTCCGAGTACACGTTCTGGGGCCAAGGCACGCAAGTCACC<br>GTCAGCAGCGCTGCCGCCGAGGTCCAGCTCGTCGAATCCGGCGGTGGTCTCGTGCAACCCGGTGGTAGCCTCCGCCTCTCCTGTGCTGCCAGCGGTCGAACGTTCTCC<br>TATAACCCCATGGGTTGGTTCCGCCAGGCTCCCGGTAAGGGCCGAGAACTCGTCGCCGCCATCTCCCGAACCGGTGGCTCCACCTATTACCCTGACAGCGTCGAAGGC<br>CGCTTCACCATTAGCCGCGACAACGCCAAACGCATGGTGTACCTCCAGATGAACTCCCTCCGCGCCGAGGATACGGCTGTCTACTATTGCGCCGCTGCCGGCGTCCGA<br>GCTGAAGATGGTCGCGTCCGAACCCTGCCTTCCGAGTATACCTTCTGGGGTCAAGGCACCCAGGTCACCGTCTCCTCCCACCACCATCACCACCACTAA
**Ozoralizumab (AGS-3)**	**SCK**	ATGATTGTGGGCATCCTCACGACCCTCGCTACGCTGGCTACGCTCGCTGCTTCCGTCCCCCTCGAAGAGCGCCAAGCTTGCTCCAGCGTGTGGGGCCAGTGTGGCGGT<br>CAAAATTGGTCCGGTCCCACGTGCTGCGCTAGCGGCAGCACGTGCGTCTACTCCAACGACTACTACAGCCAGTGTCTCCCCGGCGCTGCTAGCAGCAGCTCCAGCAC<br>CCGAGCTGCTTCCACCACGAGCCGAGTCAGCCCTACGACCTCCCGAGACAAGCGCGAGGTCCAACTGGTCGAATCCGGTGGCGGTCTCGTCCAACCCGGCGGTAGC<br>CTCCGACTCAGCTGCGCTGCCTCCGGCTTTACCTTCTCCGACTACTGGATGTACTGGGTGCGCCAAGCTCCCGGCAAGGGTCTCGAATGGGTCAGCGAGATCAACACG<br>AATGGCCTCATCACGAAATACCCCGACTCCGTCAAGGGCCGCTTCACGATCAGCCGCGACAACGCCAAGAACACGCTCTATCTCCAGATGAACAGCCTCCGCCCCGA<br>GGATACCGCTGTCTACTACTGTGCCCGCTCCCCTAGCGGCTTTAACCGAGGCCAAGGCACGCTGGTCACCGTGAGCAGCGGCGGTGGCGGTAGCGGCGGTGGTTCCG<br>AGGTGCAGCTCGTCGAGTCCGGTGGCGGCCTCGTGCAGCCCGGCAACTCCCTCCGCCTCAGCTGTGCCGCTTCCGGTTTCACGTTTTCCAGCTTTGGCATGAGCTGGG<br>TCCGACAAGCCCCCGGCAAGGGTCTGGAGTGGGTGAGCAGCATCAGCGGCAGCGGCTCCGATACGCTGTACGCCGATAGCGTCAAAGGCCGATTTACCATCTCCCGC<br>GATAACGCCAAGACCACGCTCTATCTGCAAATGAACTCCCTCCGACCCGAAGATACGGCCGTGTACTACTGTACGATCGGTGGTTCGCTCAGCCGCTCCTCCCAAGGC<br>ACCCTCGTCACGGTCTCCAGCGGTGGTGGTGGCTCCGGTGGTGGCAGCGAGGTCCAGCTCGTGGAATCCGGCGGCGGTCTGGTCCAGCCCGGCGGTTCGCTCCGACT<br>GAGCTGCGCTGCTTCCGGCTTTACGTTCTCCGATTACTGGATGTATTGGGTCCGACAAGCCCCCGGCAAAGGTCTGGAGTGGGTCTCCGAGATTAACACCAACGGCCT<br>CATCACCAAGTACCCCGACAGCGTGAAGGGTCGCTTTACGATCTCCCGCGACAATGCCAAGAACACCCTCTACCTCCAAATGAATTCCCTCCGCCCCGAGGACACCGC<br>CGTCTACTATTGCGCCCGATCCCCTAGCGGTTTTAACCGCGGCCAAGGTACGCTCGTCACGGTGAGCAGCCATCACCACCACCATCACTAA

Table S5 Constructed expression cassettes and primer pairs

Plasmid		Homologous region	Promoter	Gene	Terminator	Maker	Homologous region	Fragment	Forward Primer(5'→3')	Reverse Primer(5'→3')	Template
pUC-V012	pUC-ΔCbh1-Pcbh1-pep1	*cbh1-5'*	*cbh1*	*pep1*	*cbh1*	*pyr4**	*cbh1-3'*	1	GATGCGCAGTCCGCGGTTGA	CAAACCAAGAACCAAACCCCCATCTCCGCGAATCT	pUC-V008
								2	CGCGGACTGCGCATCATGCAGACCTTTGGAGCTTT	TTTCGCCACGGAGCTTTATTTCTGAGCCCAGCCCA	pUC-V010
								3	AGCTCCGTGGCGAAAGCCTG	TTGGTTCTTGGTTTGGAGGG	pUC-V007
pUC-V013	pUC-ΔCbh1-Pcbh1-gla1	*cbh1-5'*	*cbh1*	*gla1*	*cbh1*	*pyr4**	*cbh1-3'*	1	GATGCGCAGTCCGCGGTTGA	CAAACCAAGAACCAAACCCCCATCTCCGCGAATCT	pUC-V008
								2	CGCGGACTGCGCATCATGCACGTCCTGTCGACTGC	TTTCGCCACGGAGCTTTACGACTGCCAGGTGTCCT	pUC-V011
								3	AGCTCCGTGGCGAAAGCCTG	TTGGTTCTTGGTTTGGAGGG	pUC-V007
pUC-V014	pUC-ΔCbh1-Pcbh1-1ZVH-SCK	*cbh1-5'*	*cbh1*	*1zvh (cbh2 SS+CBD+Kex2 linker)*	*cbh1*	*pyr4**	*cbh1-3'*	1	GATGCGCAGTCCGCGGTTGA	CAAACCAAGAACCAAACCCCCATCTCCGCGAATCT	pUC-V008
								2	CGCGGACTGCGCATCATGATCGTCGGCATTCTCAC	TTTCGCCACGGAGCTTTAGTGATGGTGGTGATGGT	AGS-1
								3	AGCTCCGTGGCGAAAGCCTG	TTGGTTCTTGGTTTGGAGGG	pUC-V007
pUC-V015	pUC-ΔCbh1-Pcbh1-1ZVH-SC	*cbh1-5'*	*cbh1*	*1zvh (cbh2 SS+CBD)*	*cbh1*	*pyr4**	*cbh1-3'*	1	GATGTCCAGCTCGTCGAAAG	GACGAGCTGGACATCGCTGGTGGTAGGGCTGACTC	pUC-V014
pUC-V016	pUC-ΔCbh1-Pcbh1-1ZVH-S	*cbh1-5'*	*cbh1*	*1zvh (cbh2 SS)*	*cbh1*	*pyr4**	*cbh1-3'*	1	GATGTCCAGCTCGTCGAAAG	GACGAGCTGGACATCAGGGACGCTAGCAGCGAGGG	pUC-V014
pUC-V017	pUC-ΔCbh1-Pcbh1-Cap-SCK	*cbh1-5'*	*cbh1*	*caplacizumab (cbh2 SS+CBD+Kex2 linker)*	*cbh1*	*pyr4**	*cbh1-3'*	1	GATGCGCAGTCCGCGGTTGA	CAAACCAAGAACCAAACCCCCATCTCCGCGAATCT	pUC-V008
								2	CGCGGACTGCGCATCATGATCGTCGGCATCCTCAC	TTTCGCCACGGAGCTTTAGTGGTGGTGATGGTGGT	AGS-2
								3	AGCTCCGTGGCGAAAGCCTG	TTGGTTCTTGGTTTGGAGGG	pUC-V007
pUC-V018	pUC-ΔCbh1-Pcbh1-Cap-SC	*cbh1-5'*	*cbh1*	*caplacizumab (cbh2 SS+CBD)*	*cbh1*	*pyr4**	*cbh1-3'*	1	GAGGTCCAACTGGTGGAGTC	CACCAGTTGGACCTCGGACGTGGTAGGGCTCACTC	pUC-V017
pUC-V019	pUC-ΔCbh1-Pcbh1-Cap-S	*cbh1-5'*	*cbh1*	*caplacizumab (cbh2 SS)*	*cbh1*	*pyr4**	*cbh1-3'*	1	GAGGTCCAACTGGTGGAGTC	CACCAGTTGGACCTCAGGGACGGAAGCAGCGAGCG	pUC-V017

pUC-V020	pUC-ΔCbh1-Pcbh1-Ozo-SCK	*cbh1-5'*	*cbh1*	*ozoralizumab (cbh2 SS+CBD+Kex2 linker)*	*cbh1*	*pyr4**	*cbh1-3'*	1	GATGCGCAGTCCGCGGTTGA	CAAACCAAGAACCAAACCCCCATCTCCGCGAATCT	pUC-V008
								2	CGCGGACTGCGCATCATGATTGTGGGCATCCTCAC	TTTCGCCACGGAGCTTTAGTGATGGTGGTGGTGAT	AGS-3
								3	AGCTCCGTGGCGAAAGCCTG	TTGGTTCTTGGTTTGGAGGG	pUC-V007
pUC-V021	pUC-ΔCbh1-Pcbh1-Ozo-SC	*cbh1-5'*	*cbh1*	*ozoralizumab (cbh2 SS+CBD)*	*cbh1*	*pyr4**	*cbh1-3'*	1	GAGGTCCAACTGGTCGAATC	GACCAGTTGGACCTCGGAGGTCGTAGGGCTGACTC	pUC-V020
pUC-V022	pUC-ΔCbh1-Pcbh1-Ozo-S	*cbh1-5'*	*cbh1*	*ozoralizumab (cbh2 SS)*	*cbh1*	*pyr4**	*cbh1-3'*	1	GAGGTCCAACTGGTCGAATC	GACCAGTTGGACCTCGGGGACGGAAGCAGCGAGCG	pUC-V020
pUC-V023	pUC-ΔCbh2-Pcbh2-Cap-SCK	*cbh2-5'*	*cbh2*	*caplacizumab (cbh2 SS+CBD+Kex2 linker)*	*cbh1*	*pyr4**	*cbh2-3'*	1	GGTGCAATACACAGAGGGTG	GGCTTTCGTGACCGGGCTTC	pUC-V009
								2	TCTGTGTATTGCACCATGATCGTCGGCATCCTCAC	CCGGTCACGAAAGCCTTGGTTCTTGGTTTGGAGGG	pUC-V017
